# Do Peers Matter? Resistance to Peer Influence as a Mediator between Self-Esteem and Procrastination among Undergraduates

**DOI:** 10.3389/fpsyg.2016.01529

**Published:** 2016-10-03

**Authors:** Bin-Bin Chen, Zeyi Shi, Yan Wang

**Affiliations:** Department of Psychology, Fudan UniversityShanghai, China

**Keywords:** procrastination, self-esteem, resistance to peer influence

## Abstract

This study examined the relationship between self-esteem and procrastination and the mediating role of resistance to peer influence (RPI) on this relationship among undergraduates. One hundred and ninety-nine Chinese undergraduate students completed the measures of procrastination, RPI, and self-esteem. Structural Equation Modeling analyses indicated that self-esteem was negatively related to procrastination, and RPI acted as a mediator of this relationship. The results suggest that the peer may be a key to understanding procrastination among undergraduates. Implications for future research and limitations of the current study are discussed.

## Introduction

Procrastination is defined as a purposive delay of an intended course of action, despite being aware of negative outcomes (Steel, 2007). Procrastination is a very prevalent phenomenon among undergraduate students (Solomon and Rothblum, 1984; Ferrari et al., 1995; Day et al., 2000). Procrastination was found to be related to maladaptive psychological and academic outcomes (Ferrari, 1994; Tice and Baumeister, 1997; Sirois et al., 2003; Sirois and Kitner, 2015). However, the causes of procrastination are not completely understood (Ferrari, 1994; Steel, 2007). A recent meta-analysis has demonstrated some possible causes of procrastination (Steel, 2007). For example, self-esteem is one of many factors which may affect procrastination because individuals with low levels of self-esteem fear failure or avoid negative consequences. In addition to motivating individuals to take actions in response to success and failure, self-esteem also serves the social function of restoring social inclusion to satisfactory levels (Leary et al., 1995). Nonetheless, it is surprising that there has been no exploration of how self-esteem influences procrastination in the social context of peers, which is an important social network for undergraduates. The purposes of the current study were to replicate the relation between self-esteem and procrastination and to expand previous literature, by investigating the mediating role of resistance to peer influence (RPI) on this relationship, in a Chinese sample of undergraduate students.

There is a wealth of literature indicating that procrastination is negatively related to self-esteem (e.g., Solomon and Rothblum, 1984; Ferrari, 1994; Beck et al., 2000; Klassen et al., 2008). Individuals with low self-esteem tend to engage in procrastination-relevant behaviors, which are triggered by aversive or difficult tasks. In most of these studies, therefore, procrastination is considered as a strategy to protect self-esteem (Solomon and Rothblum, 1984; Flett et al., 1995; Klassen et al., 2008).

Although previous research which focused on self-esteem for explaining procrastination was useful, little attention has been paid to the function of self-esteem itself and the social pattern of procrastination. According to the Sociometer Theory (Leary et al., 1995; Leary and Baumeister, 2000; Kirkpatrick and Ellis, 2001), self-esteem may be an adaptive psychological mechanism, designed to monitor social inclusion or acceptance in social groups and to motivate individuals to take actions to decrease the possibility of being excluded or rejected. Furthermore, research has identified being socially active as one of the most common patterns underlying procrastination (Day et al., 2000). In light of this, examination of procrastination should be beyond the intra-personal domain. From these perspectives, the relationships between self-esteem and procrastination should be examined in the social, inter-personal context. One of the most important inter-personal contexts for students in college may be their peer group (Collins and Repinski, 1994; Dennis et al., 2005; Lu, 2014). As a result, there is little reason to believe that the relationships between self-esteem and procrastination are independent of peer effects.

The literature about peer influence as a mediator for the relationships between self-esteem and behavior is still quite recent. One significant mediating process was found in the relationships between self-esteem and problem behaviors (DuBois and Silverthorn, 2004). Specifically, youth with lower level of self-esteem were susceptible to deviant peer associations, which, in turn, were linked to higher levels of problem behavior.

Based on the extant literature, RPI appears to act as a mediator in the relationship between self-esteem and procrastination. RPI is defined in terms of an individual’s level of susceptibility to peer pressure (Steinberg and Monahan, 2007). Low self-esteem causes undergraduates to be particularly sensitive in perceiving the threats of peer rejection. In order to be accepted by their peers, undergraduates with low self-esteem may be more likely to be influenced by their peers to satisfy their needs, and, as a consequence, they may have to stop or postpone their own work or the task which they are engaged in.

Previous research has shown that lower levels of self-esteem were related to higher susceptibility to peer pressure (Bukowski et al., 2008) and to an increased possibility of the involvement in gangs with a low level of membership (Dmitrieva et al., 2014). Therefore, it suggests that low self-esteem would be related to lower levels of RPI.

In addition, peers are considered as an important source of influences on an individual’s behaviors and performance. Although no research has examined the relationships between RPI and procrastination, RPI has recently been recognized as a variable that affects impulsivity, which was considered as a correlate of procrastination (Gustavson et al., 2014). For example, the presence of peers increases the impulsivity of different aspects, including the engagement in risk taking (Gardner and Steinberg, 2005; Chein et al., 2011) and the value of immediate reward (O’Brien et al., 2011). Based on the aforementioned previous literature, it is reasonable to predict that RPI might be also related to procrastination.

### The Present Study

**FIGURE 1:**
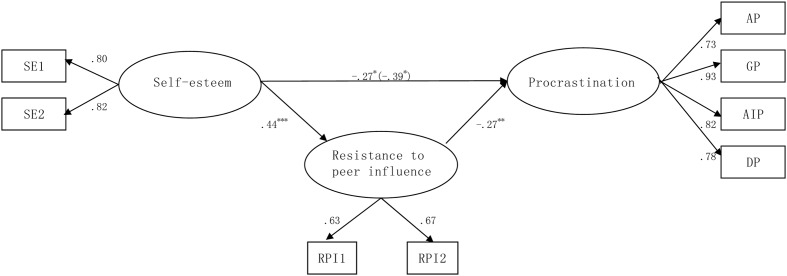
**The finalized model.** Factor loadings are standardized. RPI1 and RPI2 are two parcels of resistance to peer influence (RPI); SE1 and SE2 are two parcels of self-esteem; AP, GP, AIP, and DP represent four measures of procrastination. For the path between self-esteem and procrastination, the value in brackets represents the direct effect of self-esteem on procrastination without inclusion of the mediator. For the path coefficients, ^∗^*p* < 0.05, ^∗∗^*p* < 0.01, ^∗∗∗^*p* < 0.001.

In summary, the main aim of the present study was to examine a mechanism through which self-esteem would influence procrastination. There were two hypotheses to be tested in the present study. First, consistent with previous research, self-esteem was hypothesized to be associated with procrastination. Second, RPI was hypothesized to be a mediator in the relationship between self-esteem and procrastination. The model is presented in **Figure 1**.

We tested the hypotheses in a sample of college students. Using Structural Equation Modeling (SEM), we employed the multiple-indicator approach to measure the three latent constructs. We used four scales (i.e., academic procrastination, general behavioral procrastination, decisional procrastination, and an adult inventory of procrastination) to measure the procrastination latent construct. We applied the parceling approach (Little et al., 2002) to create multiple indicators from the RPI Scale and Rosenberg Self-Esteem Scale and, thus, to measure the RPI and self-esteem latent constructs, respectively. We relied both on the overall model fitness statistics and significance tests of specific paths to examine the direct association between self-esteem and procrastination and the indirect association between these two constructs through the mediation of RPI.

## Materials and Methods

### Participants and Procedure

One hundred and ninety-nine undergraduate students (38 males, 161 females; mean age = 19.30 years, *SD* = 1.11) were recruited from introductory psychology classes in a university located in Eastern China. Participants completed self-report measures as part of a larger online study, initiated by an evolutionary perspective on procrastination project. These measures have previously been used among Chinese undergraduates with satisfactory reliability and validity (Liu et al., 2014; Chen and Chang, 2016).

### Measures

#### Academic Procrastination (AP) Scale

The AP scale consists of six areas of academic functioning (e.g., “writing for an exam”) (Solomon and Rothblum, 1984). Participants were asked to indicate on a 5-point scale the degree to which they procrastinated on these tasks (1 = “never” to 5 = “always”). Cronbach’s alpha was 0.85 in the current study.

#### General Behavioral Procrastination (GP) Scale

The GP scale measures an individual’s tendencies in procrastination across a variety of delay tasks (e.g., “mailing a letter”) on a 5-point Likert scale (1 = “strongly disagree” to 5 = “strongly agree”) (Lay, 1986). Cronbach’s alpha was 0.84 in the current study.

#### Adult Inventory of Procrastination (AIP) Scale

The AIP scale was used to measure the behavioral tendency to delay in beginning or completing tasks (Ferrari et al., 1995). It consists of 15 items (e.g., “I don’t get things done on time”; α = 0.78). Participants were asked to respond to these statements using a 5-point Likert scale (1 = “strongly disagree” to 5 = “strongly agree”).

#### Decisional Procrastination (DP) Scale

The DP scale was used to measure an individual’s purposive delay in making decisions by doing other tasks (Mann, 1982). It consists of five items (e.g., “I delay in making decisions until it is too late”; α = 0.85). Participants were asked to respond to these statements using a 5-point Likert scale (1 = “strongly disagree” to 5 = “strongly agree”).

#### Resistance to Peer Influence Scale

The RPI scale consists of 10 items to assess an individual’s propensity to resist the influence of his or her peers (Steinberg and Monahan, 2007). In order to minimize the influence of social desirability response biases, each item contains two opposing statements with the “Some people… BUT Other people…” format. Participants were asked to designate which statement was more like themselves and to indicate the degree of the item’s applicability. For example, participants read a statement such as “Some people go along with their friends just to keep their friends happy, BUT Other people refuse to go along with what their friends want to do, even though they know it will make their friends unhappy.” They were asked which statement was true about themselves, and whether it was “really true” or “sort of true.” Each item was scored from 1 to 4 with 1 indicating “really true for me” on the first statement and 4 indicating “really true for me” on its opposite statement. All items were averaged to generate one total score, with higher scores indicating greater RPI. Cronbach’s alpha was 0.55 in the current study.

#### Rosenberg Self-Esteem (RSE) Scale

The RSE scale was used to assess global self-esteem (Rosenberg, 1965). It comprises 10 items (e.g., “I feel that I have a number of good qualities”; α = 0.85). Items were presented on a 4-point Likert scale ranging from 1 = “strongly disagree” to 4 = “strongly agree”. The scores across all items were summed to generate a total score, where higher scores indicated higher levels of self-esteem.

## Results

### Descriptive Analyses

**Table 1** shows all means, standard deviations, and intercorrelations. All correlations were in the expected direction. All procrastination variables were correlated with each other. Procrastination variables were negatively related to both RPI and self-esteem. Finally, RPI was positively related to self-esteem.

**Table 1:** Descriptive statistics and correlations of observed variables.

Variables	1	2	3	4	5	6
(1) Academic procrastination	-					
(2) General behavioral procrastination	0.68^∗∗∗^	-				
(3) Adult inventory of procrastination	0.59^∗∗∗^	0.76^∗∗∗^	-			
(4) Decisional procrastination	0.58^∗∗∗^	0.72^∗∗∗^	0.62^∗∗∗^	-		
(5) Resistance to peer influence	-0.17^∗^	-0.26^∗∗∗^	-0.21^∗∗^	-0.27^∗∗∗^	-	
(6) Self-esteem	-0.26^∗∗∗^	-0.30^∗∗∗^	-0.28^∗∗∗^	-0.34^∗∗∗^	0.29^∗∗∗^	-
*M*	15.32	53.67	38.54	14.98	2.62	27.10
*SD*	5.18	10.26	7.30	4.31	0.37	4.47


*^∗^*p* < 0.05; ^∗∗^*p* < 0.01; ^∗∗∗^*p* < 0.001.*

### Structural Equation Modeling

To test the relationships among RPI, self-esteem and procrastination, SEM was conducted using Mplus 7.0 (Muthén and Muthén, 2012). To correct for inflated measurement error and increase the stability of the indicators in the model, two item parcels were created for each of RPI and self-esteem factors (Parceling is widely used in SEM studies; Bandalos and Finney, 2001; Little et al., 2002). Evaluation of the fit of the model was carried out on the basis of inferential goodness-of-fit statistics (χ^2^), and a number of other indices, including the comparative fit index (CFI), the root-mean-square error of approximation (RMSEA), and the Standardized Root Mean Square Residual (SRMR). Values close to or greater than 0.95 are desirable on the CFI, while the RMSEA and SRMR should preferably be less than or equal to 0.06 (Hu and Bentler, 1999; Millsap, 2002).

The structural models were then developed to test the two hypotheses. First, the hypothesis that self-esteem has a direct effect on procrastination was tested. The result indicated that self-esteem was negatively related to procrastination (β = -0.39, *p* < 0.001). The model fits the data well, χ^2^ (8) = 6.15, *p* > 0.05, RMSEA = 0.00, CFI = 1.00, SRMR = 0.02.

Second, RPI was added into the model to test the hypothesis that RPI mediated the associations between self-esteem and procrastination. To examine this hypothesis, two structural models were tested. The baseline model, Model 1, is a partial mediation model that includes the paths from self-esteem to RPI, RPI to procrastination, and self-esteem to procrastination. Model 2 is a full mediation model in which the direct path from self-esteem to procrastination was omitted. Results demonstrated that Model 1 fits the data well, χ^2^ (17) = 12.91, *p* > 0.05, RMSEA = 0.00, CFI = 1.00, SRMR = 0.02. When the direct path from self-esteem to procrastination was removed in Model 2, the model also fits the data well, χ^2^ (18) = 18.80, *p* > 0.05, RMSEA = 0.02, CFI = 1.00, SRMR = 0.05. Chi-square difference tests were conducted to compare the two nested models. χ^2^ in Model 2 was increased significantly [Δχ^2^ (1) = 5.89, *p* < 0.05]. This indicated that, although Model 2 had better simplicity than Model 1, the model fit worsened significantly. Therefore, Model 1, which had a superior fit to offset the reduction of parsimony by 1, was selected as the optimal model. **Figure 1** shows the path coefficients of the final selected model^1^^,^^2^.

The significance of the indirect effects of self-esteem on procrastination through RPI was tested using the Bootstrap estimation procedure. We generated 1000 bootstrapping samples from the original data set (*N* = 199) by random sampling. The standardized indirect effect of self-esteem on procrastination through RPI was significant [point estimate = -0.12, *SE* = 0.06, 95% CI = (-0.23; -0.01)]. The effect size estimate for the indirect effect of self-esteem is *R*_m_ = 1.76, the ratio of the indirect effect to the direct effect (Sobel, 1982; Preacher and Kelley, 2011), which indicates that the indirect effect of self-esteem on procrastination is approximately 1.76 times the size of the direct effect. Therefore, the mediating effects of RPI, proposed in the second hypothesis, was supported. Results indicated that self-esteem was positively related to RPI, and RPI was negatively related to procrastination.

## Discussion

The present study aimed at testing the relationship between self-esteem and procrastination and the mediating role of RPI on this relationship. In line with the expectations, the direct effect of self-esteem on procrastination was significant. That is, undergraduates with high levels of self-esteem were less likely to procrastinate.

In addition, consistent with the hypothesis, the mediating effect of RPI on the relationships between self-esteem and procrastination was significant. In other words, self-esteem indirectly and negatively affected procrastination through RPI. Undergraduates with higher levels of self-esteem might be more likely to resist the influence of their peers, which may contribute to a decrease in their level of procrastination. Although previous studies explained that procrastination is the result of the protection of self-esteem (Solomon and Rothblum, 1984; Flett et al., 1995; Klassen et al., 2008), this research emphasizes that peer relations may be a key to understanding procrastination among undergraduates. For individuals with low levels of self-esteem, successfully maintaining their connections to peers comes at a cost. That is, low self-esteem may motivate individuals to take action (e.g., task delay) to restore social inclusion to satisfactory levels. Therefore, when faced with psychosocial factors, such as peer influence and stress, the procrastination of undergraduates with low self-esteem may reflect the products of their tradeoff between efforts invested in seeking peer inclusion and efforts invested in completing one’s own task.

The present study contributes to the literature in several ways. First, it extends our understanding of procrastination in the social inter-personal domain. Most of previous studies focused on procrastination in the intra-personal domain such as personality, self-esteem, and self-regulation (Steel, 2007). The present research provided the first evidence that peer role might be related to procrastination. This new finding not only helps construct new models of procrastination (Steel, 2010), but also develops fresh approaches to reduce procrastination (e.g., increasing the likelihood of RPI).

Second, procrastination was measured with multiple questionnaires relating to different areas, such as academic, decisional, and behavioral procrastination. Different areas of procrastination may have different psychological meaning and characteristics (e.g., Ferrari, 1992; Díaz-Morales et al., 2006). The present research, including multiple questionnaires, may provide richness in the assessment of the associations addressed in the present study.

Third, this study goes beyond previous research about procrastination, by testing the potential relationships at the level of a latent variable rather than a manifest variable. SEM with latent variables has one of important advantages—it allows controlling for measurement errors in the analysis (Anderson and Gerbing, 1988).

### Limitations and Future Direction

The findings in the present study must be interpreted cautiously because of several limitations. First, it is important to bear in mind that the findings apply to undergraduates in China. The Chinese have been persistently assumed to be a distinct entity for cross-cultural research because their value system differs from most Western societies in the emphasis that is placed on collective harmony and relatedness (Ho, 1986; Markus and Kitayama, 1991; Oyserman et al., 2002). Therefore, Chinese undergraduates may be more likely to be influenced by their peers. A future direction of high priority is to examine whether these effects remain in individualistic societies.

Second, causal links between the study variables could not be determined given the correlational nature of the data with a cross-sectional design. All variables were assessed simultaneously, therefore, it lacked the time sequence relating self-esteem (i.e., the cause) to RPI (i.e., the mediator) and procrastination (i.e., the effect). The preferred solution to this problem is to use a longitudinal design to confirm the proposed relationships. In particular, future studies should include prior measures of self-esteem, RPI, and procrastination in the model, in order to allow for autoregressive effects and time lags in proposed causal relationships (Maxwell and Cole, 2007).

Finally, the present study focused on inter-personal aspects when examining the relationships between self-esteem and procrastination. We did not examine the role of intra-personal characteristics, such as self-control. In this respect, it would be interesting to examine, in future, whether self-control, as a high order variable, would be a mediator for the relationships between self-esteem and procrastination. Also it is possible that self-control may be a confound factor which may influence the association patterns of the study. Therefore, future studies should include self-control in the analysis to test whether these association patterns persist after adjustment for this potential confounding factor.

## Conclusion

This study is the first, to our knowledge, to reveal the role that RPI plays in the link between self-esteem and procrastination. Continuing to examine the causes of procrastination through the perspectives of inter-personal relations, such as friendship or romantic relationships, could enhance our understanding of the nature and origins of procrastination.

## Author Contributions

B-BC developed the study concept and design, tested and data collection, B-BC, ZS, and YW analyzed and interpreted the data, and drafted the manuscript.

## Conflict of Interest Statement

The authors declare that the research was conducted in the absence of any commercial or financial relationships that could be construed as a potential conflict of interest.

## Footnotes

1 We also tested three alternative models by not specifying hypothesized order of the variables (e.g., resistance to peer pressure → self-esteem → procrastination). The models were much poorer fit with the data compared to the hypothesized model [Δχ^2^ (1) = 5.49, 5.49, and 5.89, *p*s < 0.05].

2 We conducted analyses through Mplus to test a possible moderating role of resistance to peer influence (RPI) on the relationship between self-esteem and procrastination. The model fit the data well, χ^2^ (29) = 23.81, *p* > 0.05, RMSEA = 0.00, CFI = 1.00, SRMR = 0.03. In addition, the main effects of self-esteem (β = -0.26, *p* < 0.05) and RPI (β = -0.30, *p* < 0.05) were significantly associated with procrastination. However, the moderating effect of RPI on the relationship between self-esteem and procrastination was not significant (β = -0.10, *p* > 0.05). Therefore, the result removed the possibility that RPI moderated the relationship between self-esteem and procrastination.
